# Supplementary material for: Only human after all? a pre-registered study on gaze behavior and humanity attributions to people with facial difference
Source: PLoS One. 2023 Dec 12;18(12):e0295617. doi: 10.1371/journal.pone.0295617 (PMC10715648; doi:10.1371/journal.pone.0295617)
Supplement: S1 File — Pilot study, full method section, and supplementary results. (DOCX) [file pone.0295617.s001.docx]

Supporting information

Article: “Only human after all? A pre-registered study on gaze behavior and humanity attributions to people with facial difference”

# S1. Pilot Study

Several research suggest a dehumanization of people with facial difference (FD) but no past research investigated it directly. This preliminary study is designed so as to select the measure that would best capture the dehumanization of people with FD, which will be used in a following main study. A great deal of research has been devoted to dehumanized perceptions (for reviews, see Haslam & Loughnan, 2014; Haslam & Stratemeyer, 2016; Kteily & Landry, 2022). Since, social desirability biases self-reported measures of discrimination against people with FD (Stone & Potton, 2014), only measures of subtle forms of dehumanization will be described below.

According to the dual model approach, dehumanized people are perceived as lacking either uniquely human (UH) characteristics (i.e., complex emotions, refinement, culture), i.e., characteristics that distinguish humans from other species, or human nature (HN) characteristics (e.g., moral sensitivity), i.e., characteristics that distinguish human beings from inert entities, or both (Haslam et al., 2005). In line with the mind perception approach, dehumanized people are perceived as lacking “mind” (Gray et al., 2007). More concretely, they are perceived as lacking either agency (i.e., capacity to do; e.g., to plan actions) or experience (i.e., capacity to feel; e.g., pain) or both.

Another complementary approach can be found in models of social evaluation. Typically, this approach postulates two main dimensions in social evaluation: the vertical (i.e., competence, agency) and the horizontal (i.e., warmth, communion) dimensions (for a review, see Koch et al., 2021). Within these models of social evaluation, previous research found relationships between the dehumanized perceptions and attributions of characteristics within the Stereotype Content Model (SCM: Fiske et al., 2002; Fiske, 2018), despite not a dehumanization model per se, SCM model (Harris & Fiske, 2006). More precisely, research found that people perceived as lacking both characteristics related to competence and warmth are often dehumanized (e.g., people with mental disability; Rasset et al., 2022).

This pilot study was conducted in order to determine whether people would dehumanize individuals with FD in minimal settings. Different approaches were combined so as to select the most appropriate one (i.e., the Dual Model, the Mind Perception, the social judgment; Abele, 2003; Fiske et al., 2002; Gray et al., 2007; Haslam et al., 2005). In line with previous research conducted with neuro-imagery (Hartung et al., 2019) and cognitive tasks (Boutsen et al., 2021), we assumed that people with FD would get less attributions of humanness-related characteristics in comparison to people without FD.

**Method**

***Participants***

Sixty-nine undergraduate students volunteered to participate in the study without any financial counterpart. Two participants were withdrawn from the study for signaling having a disfigurement. The remaining 67 participants comprised 44 females (mean age = 20.34 years; *SD* = 2.11). Since no previous study investigated a possible dehumanization of people with FD, we based the sample size of this study on a minimum number of 30 exploitable data per condition. A sensitivity analysis for *F* tests for repeated measures ANOVA with within-between interactions was conducted using the G*Power software package (Faul et al., 2009) based on a sample size *N* = 60, a standard parameter of α = .05, and a power 1 – ß = .80. This analysis revealed that we could detect a small effect size (η^2^ = .03).

***Measures*** (see the details of each scale in Table S1)

**Dual Model** **Questionnaires** (based on the work of Haslam et al., 2008). Since this model provides two distinct measures (i.e., animalistic dehumanization and mechanistic dehumanization), they should be dealt with in two distinct questionnaires. The items presented hereafter were selected based on an unpublished pretest (*N* = 556) which was not focused on people with disfigurement but was conducted with a view to confirm the dimension of each word in their French translation following the work of Haslam and colleagues (2008). Participants were asked to indicate the extent to which they estimated the person on the picture had each trait on a scale ranging from 1 (‘not at all’) to 7 (‘totally’).

***Subtle Animalistic Dehumanization Questionnaire***. Three uniquely human traits (UH; Cronbach’s α = .70) and three non-uniquely human traits (non-UH; α = .70) were presented to the participants.

***Subtle Mechanistic Dehumanization Questionnaire***. Three human nature traits (HN; α = .85) and three non-human nature traits (non-HN; α = .74) were proposed to participants.

**Mind Perception Questionnaire** (Gray et al., 2007). The capacity of agentism was measured with seven items (α = .89) and the capacity of experience was measured with twelve items (α = .93). Participants were asked to indicate the extent to which they estimated the person on the picture had the capacity of each item on a scale ranging from 1 (‘not at all capable’) to 7 (‘totally capable’).

**Social Judgment Questionnaire** (Abele & Wojciszke, 2014; Carrier et al., 2014; Fiske et al., 2002; Wojciszke & Abele, 2008)*.* Competence, warmth, agency, and communality were assessed based on a measure including four items reflecting trait of competence (α = .91), four items reflecting trait of warmth (α = .91), four items reflecting trait of agentism (α = .78), and four items reflecting trait of communality (α = .76). Participants were asked to indicate the extent to which they estimated the person on the picture had each trait on a scale ranging from 1 (‘not at all’) to 7 (‘totally’).

**Table S1.** Detail of all word stimuli of each Dehumanization scale in English (and French).

| **Dual Model** | | | |
| --- | --- | --- | --- |
| **UH** | **Non UH** | **HN** | **Non HN** |
| Rational (Rationnelle)  Cultivated (Cultivée)  Broadminded (Ouverte d’esprit) | Instinctive  (Instinctive)  Impulsive (Impulsive)  Responsive (Réactive) | ﻿Comprehensive (Compréhensive)  Emotionally sensitive (Sensible émotionnellement)  Morally sensitive (Sensible moralement) | Cold (Froide)  Rigid (Rigide)  Logical (Logique) |

| **Mind perception** | |
| --- | --- |
| **Agentism - Capacity of…** | **Experience - Capacity to feel/have…** |
| Morality (Agir moralement)  Self-control (Se contrôler)  Emotion recognition (Reconnaître des émotions)  Memory (Mémoriser)  Communication (Communiquer)  Planning (Planifier)  Thought (Penser) | Hunger (Faim)  Pride (Fierté)  Pleasure (Plaisir)  Desire (Envie)  Shame (Honte)  Pain (Douleur)  Consciousness(Conscience)  Personality (Personnalité)  Rage (Colère)  Fear (Peur)  Joy (Joie)  Embarrassment (Embarras) |

| **Social judgment** | | | |
| --- | --- | --- | --- |
| **Competence** | **Warmth** | **Agentism** | **Communality** |
| Competent (Compétente)  Efficient (Efficace)  Capable (Capable)  Intelligent (Intelligente) | Warm (Chaleureuse)  Likeable (Aimable)  Sympathetic (Sympathique)  Friendly (Amicale) | Self-confident (Confiante en elle)  Forward-looking (Prévoyante)  Assertive (Affirmée)  Ambitious (Ambitieuse) | Tolerant (Tolérante)  Honest (Honnête)  Loyal (Loyale)  Caring (Bienveillante) |

***Procedure***

Participants were recruited directly on the campus. They all volunteered to participate and provided an informed consent. Participants were tested individually in a focus room on a computer. Participants were naïve to the hypotheses of the experiment. They were randomly assigned to one of the two experimental conditions. Participants in the “control condition” were invited to form an impression on a picture of a face of a woman in its original version. For participants in the “disfigurement condition” received the same instruction but the woman displayed a burn-like scar on one cheek (see Fig 1). ﻿The faces used were taken from a dataset from previous experiments (Rasset et al., 2022). For all participants, the picture of the woman was repeatedly presented above each questionnaire, which were presented in a random order. At the end of the completion, participants were asked to report socio-demographical information. They were then fully debriefed and thanked.

**Fig 1.** Faces without and with disfigurement used in Pilot Study.


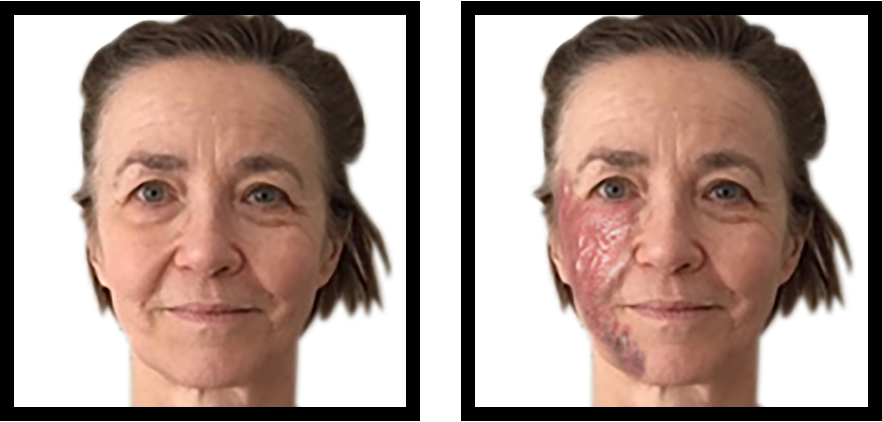


***Statistical Analysis***

A mixed design was employed with the Type of face as a between-subject variable (original vs. disfigured) and Dimensions of each measure as within-subject variable. Statistical analyses were performed using Jasp software (Jasp Team, 2021).

Within the dual model, we assumed that the person with FD should get higher attributions of UH and/or NH characteristics but equal or less attributions of non-UH and/or non-NH characteristics in comparison to the person in her original version. Within the mind perception model, we assumed that the person with FD should get higher attributions of agency and/or experience-related characteristics in comparison to the person in her original version. Within the SCM, we assumed that the person with FD should get higher attributions of competence and/or agency and/or warmth and/or communality related characteristics in comparison to the person in her original version.

**Results**

**Table S2.** Means (and standard deviations) of all measures.

|  | **Mind perception** | | **Dual model** | | | | **SCM** | | | |
| --- | --- | --- | --- | --- | --- | --- | --- | --- | --- | --- |
| **Type of face** | *Agency* | *Exp.* | *UH* | *HN* | *Non UH* | *Non HN* | *Comp.* | *Warmth* | *Agency* | *Commu.* |
| **Original** | 6.15  (0.80) | 6.16  (0.95) | 5.43  (0.98) | 5.59  (1.25) | 4.41 (1.07) | 4.05 (1.34) | 5.68 (0.97) | 5.60 (1.14) | 5.15 (1.33) | 5.46  (0.98) |
|  |  |  |  |  |  |  |  |  |  |  |
| **With FD** | 6.13  (1.02) | 6.20  (0.90) | 5.51  (1.03) | 5.82  (1.13) | 4.66 (1.37) | 4.03 (1.54) | 5.94 (1.20) | 5.96 (0.96) | 5.07 (1.15) | 5.83 (0.91) |

*Note.* FD = Facial Difference, Exp. = experience, UH = Uniquely Human, HN = Human Nature, Comp. = Competence, Commu. = Communality

***Dual Model***

Two 2 (Type of face: original, disfigurement) X 2 (Dimension: UH or HN, non-UH or non HN) repeated measures ANOVAs were run.

For the subtle animalistic dehumanization questionnaire, only a main effect of Dimension emerged, revealing that the participants attributed more UH traits to the targets (*M* = 5.47, *SD* = 1.00) than non-UH traits (*M* = 4.56, *SD* = 1.24; *F*(1, 65) = 61.87, *p* < .001, η^2^_G_ = .14). Neither the main effect of condition nor the interaction of both independent variables was significant (*F*s < 1).

For the subtle mechanistic dehumanization questionnaire, only a main effect of Dimension emerged, revealing that the participants attributed more HN traits to the targets (*M* = 5.72, *SD* = 1.18) than non-HN traits (*M* = 4.04, *SD* = 1.44; *F*(1, 65) = 72.51, *p* < .001, η^2^_G_ = .29). Neither the main effect of Type of face nor the interaction of both independent variables was significant (*F*s < 1; see Table S2 for details).

***Mind Perception***

A 2 (Type of face: original, disfigurement) X 2 (Dimension: capacity of agentism, capacity of experience) repeated measures ANOVAs were run.

Neither main effects from - nor interaction effects between the two independent variables were evidenced (*F*s < 1; see Table S2 for details).

***Social Judgment***

A 2 (Type of face: original, disfigurement) X 4 (Dimension: competence trait, warmth trait, agentism trait, communality trait) repeated measures ANOVAs were run. Greenhouse-Geisser corrections was used to correct the analysis that violated the assumption of sphericity.

Only a main effect of Dimension emerged (*F*(2.20, 142.85) = 15.18, *p* < .001, η^2^_G_ = .06), showing that participants attributed fewer agentism trait to the targets (*M* = 5.11, *SD* = 1.23) than communality trait (*M* = 5.66, *SD* = 0.96, *p*_Bonferroni_ < .001), competence trait (*M* = 5.82, *SD* = 1.10, *p*_Bonferroni_ < .001), or warmth trait (*M* = 5.79, *SD* = 1.06, *p*_Bonferroni_ < .001). The three latter were not significantly differently attributed (all *p*_Bonferroni_*s* > .98). Neither main effect of Type of face (*F*(1, 65) = 1.02, *p* = .32) nor interaction effect (*F*(2.20, 142.85) = 1.64, *p* = .19) were evidenced (see Table S2 for details).

**Discussion**

Together, these results do not support the hypothesis of a dehumanization of people with FD, nor a devaluation on any social judgment dimension.

Nevertheless, this study has several limitations. Since a single face was presented, the (absence of) results may be narrowed to this specific face. On one side, our design may have seemed too minimalist, and the possibility to realize a relative judgment while comparing different faces may have given different results. On the other side, most people are aware of the proscriptive norm that enjoins people not to ‘judge a book by its cover’, and our results may have also been biased by social desirability (Stone & Potton, 2014).

We decided not to replicate this pilot study and to still run the eye-tracking study, while taking a few precautions. First, we decided to use a within-subject design, and two sets of faces. In this way, we aimed 1) to control for any effect of face and 2) to facilitate social judgment while allowing comparisons. Second, we decided to use a hetero-perception formulation to reduce social desirability (for a similar procedure, see Louvet et al., 2009).

**References**

Abele, A. E. (2003). The Dynamics of Masculine-Agentic and Feminine-Communal Traits: Findings from a Prospective Study. *Journal of Personality and Social Psychology*, *85*(4), 768–776. https://doi.org/10.1037/0022-3514.85.4.768

Abele, A. E., & Wojciszke, B. (2014). Communal and Agentic Content in Social Cognition. In *Advances in Experimental Social Psychology* (1st ed., Vol. 50, pp. 195–255). Elsevier Inc. https://doi.org/10.1016/B978-0-12-800284-1.00004-7

Carrier, A., Louvet, E., & Rohmer, O. (2014). Compétence et agentisme dans le jugement social. *Revue Internationale de Psychologie Sociale*, *27*(1), 95–125.

Faul, F., Erdfelder, E., Buchner, A., & Lang, A.-G. (2009). Statistical power analyses using G*Power 3.1: Tests for correlation and regression analyses. *Behavior Research Methods*, *41*, 1149–1160.

Fiske, S. T., Cuddy, A. J. C., Glick, P., & Xu, J. (2002). A model of (often mixed) stereotype content: Competence and warmth respectively follow from perceived status and competition. *Journal of Personality and Social Psychology*, *82*(6), 878–902. https://doi.org/10.1037/0022-3514.82.6.878

Gray, H. M., Gray, K., & Wegner, D. M. (2007). Dimensions of Mind Perception. *Science*, *315*(5812), 619–619. https://doi.org/10.1126/science.1134475

Haslam, N., Bain, P., Douge, L., Lee, M., & Bastian, B. (2005). More human than you: Attributing humanness to self and others. *Journal of Personality and Social Psychology*, *89*(6), 937–950. https://doi.org/10.1037/0022-3514.89.6.937

Haslam, N., Loughnan, S., Kashima, Y., & Bain, P. (2008). Attributing and denying humanness to others. *European Review of Social Psychology*, *19*(1), 55–85. https://doi.org/10.1080/10463280801981645

Jasp Team. (2021). *JASP (Version 0.16)*.

Louvet, E., Rohmer, O., & Dubois, N. (2009). Social Judgment of People with a Disability in the Workplace. *Swiss Journal of Psychology*, *68*(3), 153–159. https://doi.org/10.1024/1421-0185.68.3.153

Rasset, P., Mange, J., & Montalan, B. (2022). Look me in the eyes! A pre-registered eye-tracking study investigating visual attention and affective reactions to faces with a visible difference. *Body Image*, *40*, 67–77. https://doi.org/10.1016/j.bodyim.2021.10.010

Stone, A., & Potton, A. (2014). Emotional Responses to Disfigured Faces: The Influences of Perceived Anonymity, Empathy, and Disgust Sensitivity. *Basic and Applied Social Psychology*, *36*(6), 520–532. https://doi.org/10.1080/01973533.2014.958491

Wojciszke, B., & Abele, A. E. (2008). The primacy of communion over agency and its reversals in evaluations. *European Journal of Social Psychology*, *38*(7), 1139–1147. https://doi.org/10.1002/ejsp.549

# S2. Full method section

**Purpose**

The aims of this research were twofold: 1) to replicate previous eye-tracking research showing a stigmatizing gaze with a focus on the FD neglecting the eyes region (Rasset et al., 2022), and 2) to determine if this stigmatizing gaze would be related to the dehumanization of people with FD. Specifically, we firstly sought to replicate former work showing that the FD is looked at for longer duration which diverts the gaze from the eyes’ region. We secondly assumed that people with FD would be denied humanness-related characteristics in comparison to people without FD. We thirdly postulated that this stigmatizing gaze would be negatively correlated with the attributions of humanness-related characteristics.

**Participants**

One hundred and thirteen participants volunteered to participate in the study without any financial counterpart. Twelve participants were withdrawn from the study because of calibration errors and four for having a disfigurement. The remaining 97 participants comprised 65 females, 30 males, and 2 non binary participants (M = 19.61 years; SD = 3.01).

G*Power (Buchner et al., 2017) was used to calculate an a priori sample size, using a standard parameter of α = .05, a power 1 – ß = .80, for a small effect of ρ = .25 (Rasset et al., 2022). This study was preregistered at https://osf.io/grytk.

**Stimuli**

The faces used in this experiment were selected from the database of Rasset and colleagues (2022). Two sets of four faces were set up. In each set, participants could see two faces with FD and two faces without; each time one face belonged to a woman, and the other to a man. For the two faces presenting a FD: one of them had a FD pattern on the left cheek, while the other had a FD on the right one. Each face was presented either in its original or in its “disfigured” condition depending on the set of face. Such precautions were taken to prevent - or at least control - for any effect of sex, specificity or lateralization of face perception.

**Measures**

***Eye-tracking indicators:***

*Total Dwell Time* (i.e., the sum of all dwell times for the one and same AOI over a trial). For each AOI, the total dwell times (in seconds) were calculated by averaging the total dwell times of all stimuli. Mean total dwell times were calculated separately for FD and original faces.

*Time to First Fixation* (i.e., the time period from entering the AOI until the first fixation is made). For each AOI, the mean time to first fixation was calculated by determining, relative to the onset of the trial, how long it took for participants to initially fixate in an AoI with lower values representing a faster orientation of the gaze. If an AoI was not fixated in the 5 s window, a value of 5 was given. For each AOI, the mean time to first fixation (in seconds) was calculated by averaging the time to first fixation of all stimuli. The mean times to first fixation were calculated separately for FD and original faces.

***Dual Model Questionnaires*** (based on the work of Haslam et al., 2008). The same items as those used in the pilot study (see Supplementary Materials) were used for UH traits (α = .74) and HN traits (α = .61). Participants had to report “the extent to which most people seeing the person on the photograph may think that the person is” the written trait on a numeric scale of 1–7 (corresponding to: 1- ‘totally disagree’, 4 – ‘neither agree nor disagree’, 7 – ‘totally agree’).

**Procedure**

All participants were recruited directly on the campus by the experimenter and volunteered to participate. Since all participants were naïve to the hypotheses of the experiment, they provided a first a priori informed consent. They were then tested individually in a focus room. Gaze was measured using a Tobii X120 Eye-tracker (SR Research Ltd.), with a sampling rate of 120 hz (for more details on the fixation filter and the calibration procedure, see Rasset et al., 2022).

First, the eye-tracker was calibrated. Then, the experimenter left the participants alone in the experiment room. Each trial consisted of the following sequence: a fixation cross in the center of the screen displayed for 2 seconds, then a face displayed for 5 seconds. Following each face presentation, participants were asked to evaluate the face with humanness-related attributes, presented one by one, in random order. Each face was presented six times in fixed random order, once for each attribute, thus bringing the total of trials to 24. Each trial lasted approximately 10 seconds.

The length and location of the participants’ visual fixations were recorded while participants were viewing each face. For that purpose, five separate nonoverlapping Areas of Interest (AoI) were manually drawn on for the eyes, nose, target cheek, other cheek, and lips on each of the face displays (based on Fincher, 2019; see Figure 1). After providing socio-demographic information, they were then fully debriefed, gave a second a posteriori informed consent and were finally thanked.

**S1 Fig. Schematic representation of the regions of interest used in the analysis. See the online article for the color version of this figure.**


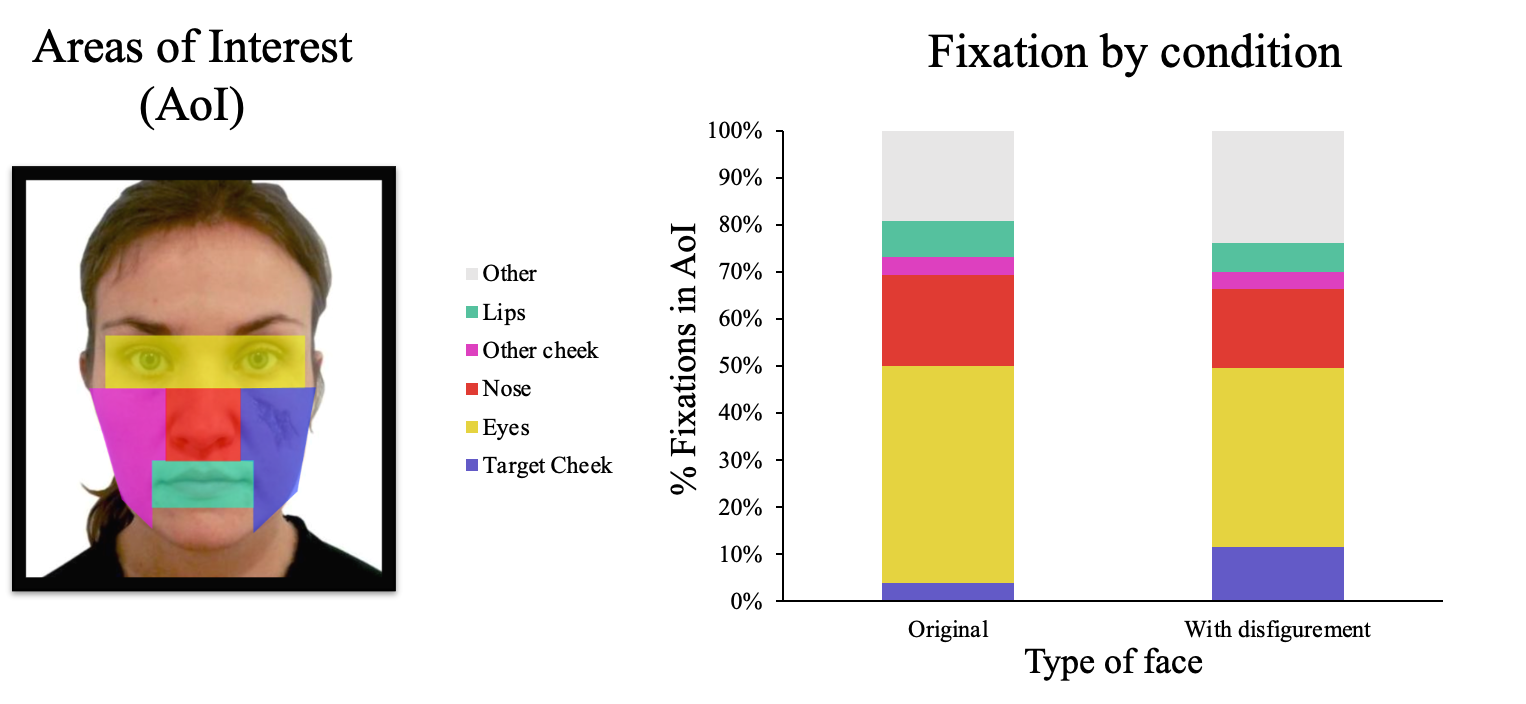


**Statistical Analysis**

First, the hypothesis of a disfigurement impact on visual attention was tested. For that purpose, a 2 (Type of face: original, wih FD) X 5 (AoI: eyes, nose, target cheek, other cheek, lips) repeated measures ANOVAs were run on each eye tracking variables. Greenhouse-Geisser corrections were used to correct the analyses that violated the assumption of sphericity. Second, the assumption of the FD’s impact on humanness-related attributes was tested. For that purpose, a 2 (Type of face: original, with FD) X 2 (Humanness-related attribute: UH, HN) repeated measures ANOVA were run. Finally, the relationships between humanity attributions and visual attention were explored through correlations and linear regressions. For that purpose, for all measures, scores were computed by considering the difference mean score of the faces with disfigurement minus the original faces mean score (for a similar procedure, see Stone & Potton, 2019). Higher (positive) scores systematically indicate greater humanness-related attributions / attention to faces with disfigurement whereas lower (negative) scores refer to greater humanness-related attributions / attention to original faces.

Statistical analyses were performed using the Jasp software (Jasp Team, 2021) and the Jamovi software (The jamovi project, 2021).

**Ethics**

Ethical approval was obtained from the local institutional review committee of the laboratory of CRFDP – University of Rouen Normandy (N° 2020–06-A). Every participant gave their informed consent to participate in the studies.

# S3. Supplementary results

**S1 Table. Means (Standard Deviations) and correlations (Bravais-Pearson’s *r*) between and of eye-tracking variables difference scores for each AoI and humanness difference scores in Pre-registered Study**

| **AoI** | **UH** | **HN** | ***M (SD)*** |
| --- | --- | --- | --- |
| **Total dwell time** |  |  |  |
| Eyes | -.08 | .15 | -0.29 (0.55) |
| Nose | .00 | -.02 | -0.09 (0.30) |
| Target cheek | -.07 | -.05 | 0.46 (0.34) |
| Other Cheek | -.14 | -.11 | -0.01 (0.16) |
| Lips | .19 | .00 | -0.03 (0.17) |
| **Time to first fixation** | | | |
| Eyes | .15 | -.10 | 0.11 (0.65) |
| Nose | -.01 | .00 | 0.17 (0.72) |
| Target cheek | .01 | .02 | -1.51 (1.04) |
| Other Cheek | .17 | .17 | 0.02 (0.67) |
| Lips | -.10 | -.08 | 0.24 (0.72) |
|  |  |  |  |
| ***M (SD)*** | -0.04 (1.01) | 0.37 (1.26) |  |

*Note*: Higher (lower) values (either) indicate positive (or negative) correlations between total dwell time and humanness attributions for disfigured faces.

**S2 Fig.** **Total dwell time (mean and standard deviation) occurring in each AoI depending on the type of face in Pre-registered study.**


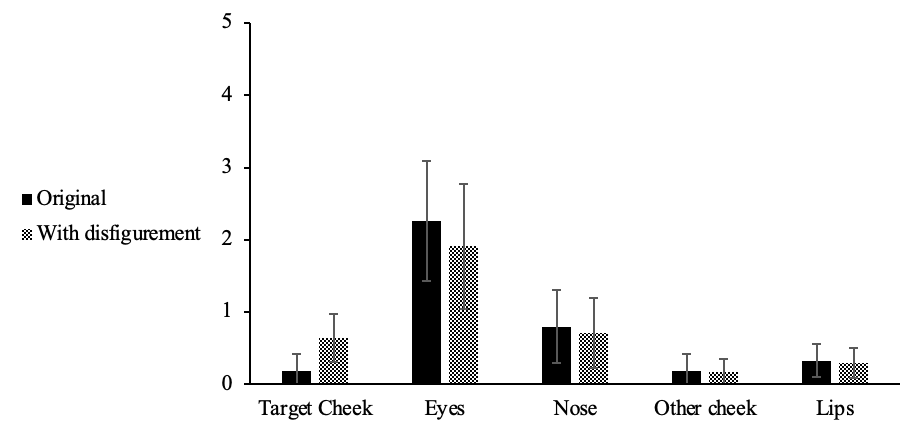


**S3 Fig. Time to first fixation (mean and standard deviation) occurring in each AoI depending on the type of face in Pre-registered study.**

**
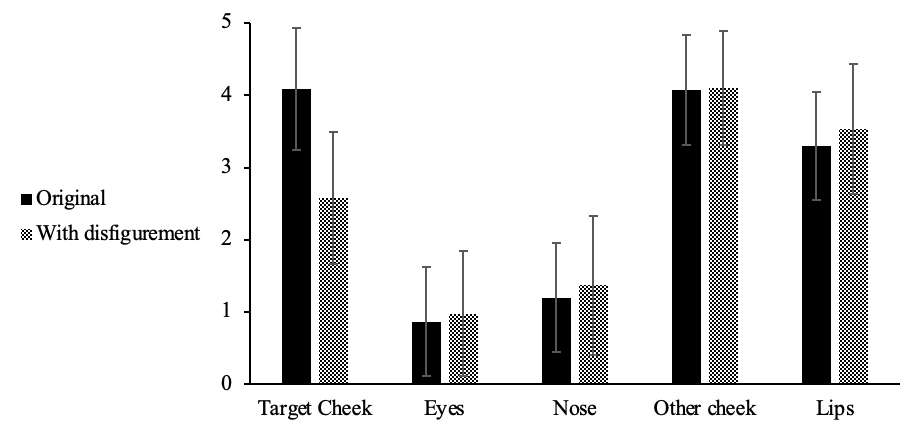
**
