# Supplementary material for: Only human after all? a pre-registered study on gaze behavior and humanity attributions to people with facial difference
Source: PLoS One. 2023 Dec 12;18(12):e0295617. doi: 10.1371/journal.pone.0295617 (PMC10715648; doi:10.1371/journal.pone.0295617)
Supplement: Attachment — Submitted filename: Response to Reviewers.docx [file pone.0295617.s002.docx]

Submission of the revised version of a paper in “PLOS ONE”

Responses to Reviewers

PLOS ONE
Ref: PONE-D-23-17359

October, 26th, 2023

# **Editor**

***1. Please ensure that your manuscript meets PLOS ONE's style requirements, including those for file naming.***

**Response:** We've made sure that the paragraphs are justified. We put an indent at the beginning of the abstract. We redid the title page. We have renamed the files as requested.

We hope this new version will meet your expectations.

**Modifications**:

- Supplementary information: We added a supplementary information section (p.17): « S1 File. Supplementary file. Pilot study, full method section, and supplementary results. »

***2. Thank you for stating in your Funding Statement […]. Please provide an amended statement that declares *all* the funding or sources of support (whether external or internal to your organization) received during this study, as detailed online in our guide for authors at*** <http://journals.plos.org/plosone/s/submit-now>.  Please also include the statement “There was no additional external funding received for this study.” in your updated Funding Statement. Please include your amended Funding Statement within your cover letter. We will change the online submission form on your behalf.

**Response:** We rectified the grant allocation. We added the statement as requested.

**Modifications**:

- Cover letter: “This study was supported by the Normandy Region [90 000 €, 2018; grant RIN tremplin 19E00851, 2019] to JM, and a grant from the Foundation Gueules Cassées [22 910 €, 2020] to PR. These funders did not play any role in the study design, data collection and analysis, decision to publish, or preparations of the manuscript. There was no additional external funding received for this study.”

3. We note that the grant information you provided in the ‘Funding Information’ and ‘Financial Disclosure’ sections do not match.

When you resubmit, please ensure that you provide the correct grant numbers for the awards you received for your study in the ‘Funding Information’ section.

**Response:** We rectified funding information.

**Modifications**:

- Cover letter: “This study was supported by the Normandy Region [90 000 €, 2018; grant RIN tremplin 19E00851, 2019] to PR, and a grant from the Foundation Gueules Cassées [22 910 €, 2020] to PR.”

4. Please review your reference list to ensure that it is complete and correct. If you have cited papers that have been retracted, please include the rationale for doing so in the manuscript text, or remove these references and replace them with relevant current references. Any changes to the reference list should be mentioned in the rebuttal letter that accompanies your revised manuscript. If you need to cite a retracted article, indicate the article’s retracted status in the References list and also include a citation and full reference for the retraction notice.

**Response:** There were shifts in references. We have corrected them. In addition, we have adapted the in-text citations to the required standards. To our knowledge, we did not include retracted papers.

**Modifications**:

- Throughout the main manuscript: We removed the name of authors for in-text citations except when we describe findings from one specific research (e.g., p.4): “Madera and Hebl (2012, 2019) [17,18] revealed that the focus on FD biases memory, which in turn fosters discrimination”.
- References: We corrected the references’ number and added missing references (e.g., p.14): « 20.	Buchner, A., Erdfelder, E., Faul, F., et al. G* Power 3.1 manual. Düsseldorf, Germany: Heinrich-Heine-Universitat Dusseldorf, 2017. ».

# **Reviewer #1**

The research is competently conducted. The eye-tracking work has been analysed carefully and the measurement of the various humanness traits looks adequate, although no evidence is reported for the validity of the French translations of the chosen traits in representing the two kinds of humanness. The authors are surely correct in interpreting the surprising attribution of greater humanness to the FD faces as being due to social desirability. Unlike the gaze behaviour, the trait ratings are slow, deliberate, reflective, and easily made to be sympathetic to people seen as victims of adversity and stigma. The authors are also correct in proposing that more immediate and less bias-prone measures of dehumanization might be used in future work (e.g., implicit measures might be beneficial here) and that perceptions of being dehumanized from the target's perspective should also be examined.

**Response:** We thank the reviewer for the compliment. Considering the HN traits, we did not go through an existing scale translation procedure as we did not find a suitable HN-scale for our study. Still, we did report evidences for the validity of the chosen traits (nevertheless unpublished). This is mentioned p.3 of our supporting information file: « The items (…) were selected based on an unpublished pretest (*N* = 556) which was not focused on people with disfigurement but was conducted with a view to confirm the dimension of each word in their French translation following the work of Haslam and colleagues (2008). Participants were asked to indicate the extent to which they estimated the person on the picture had each trait on a scale ranging from 1 (‘not at all’) to 7 (‘totally’). » This pretest was conducted among French people who were asked to evaluate people with physical or mental disability. Please note that this NH scale was then used in a published study:

Rasset, P., Montalan, B., Mauny, N., Boudjemadi, V., & Mange, J. (2022). Not All ‘Intouchables’: Variations in Humanness Perceptions between Physical and Mental Disability. *International Review of Social Psychology*, *35*(1), 7.

1. it is not clear in the main study how the HU and HN measures have been constructed. The analysis seems to have been done differently than in the pilot study, where means for HN and non-HN (and HU and non-HU) traits are presented. No means for the non-HN and non-HU traits are reported in the main study, and when the authors write "participants attributed more HN traits to faces with a disfigurement" it is not clear whether they are referring to the three HN traits or to some combination of the HN and the non-HN traits. Were single scales developed for the main study by reverse-scoring the "non" traits or was some other method employed?

**Response:** In the main study, analyses have been done differently than in the pilot study. After pilot study, since dimensions from the dual model displayed good internal consistency and allowed us to differentiate between two types of dehumanization (i.e., animalistic and mechanistic), we decided to focus on this scale instead of the mind perception or the social judgment scales. Moreover, since 1) we decided to use a within-subject design in the main study (we were more interested in the comparison between faces with and without disfigurement rather than in the absolute dehumanization of each target, i.e., UH vs non UH and NH vs. non NH), and 2) we did not want to use too many stimuli (a long-lasting experimental procedure would have been likely to exhaust participants), we selected UH- and HN-traits only and neither non-UH nor non-HN traits.

Thus, when we wrote « participants attributed more HN traits to faces with a disfigurement » we compare attributions of HN traits to faces with a disfigurement to faces without a disfigurement.

**Modifications**:

- Procedure, material and measure: In the text, we added « only » to highlight that we were using only UH and NH traits (and not non UH and non NH traits). (p.6): “Based on a pilot study (see supporting information), only UH traits (α = .74) and HN traits (α = .61) were selected.”
- Supplementary materials - S1: In the conclusion of the pilot study, we highlighted that we would not be using non UH and non NH traits (p.8-9): « Finally, since dimensions from the dual model displayed good internal consistency and allowed us to differentiate between two types of dehumanization (i.e., animalistic and mechanistic), we decided to focus on this scale instead of the mind perception or the social judgment scales. We decided to use only UH and NH traits to avoid exhausting participants. »
- Supplementary materials - S2: In the full method section, we provided rationale for not using non UH and non HN traits (p.13): "In contrast to pilot study, since 1) we decided to use a within-subject design (we were more interested in the comparison between faces with and without disfigurement rather than in the absolute dehumanization of each target, i.e., UH vs non UH and NH vos non NH), and 2) we did not want to use too many stimuli (a long-lasting experimental procedure would have been likely to exhaust participants), we selected UH- and HN-traits only and neither non-UH- nor non-HN traits. »

2. It is possible that the higher HN ratings for the FD faces was due not (or not only) to socially desirable responding but to the specific traits used to measure HN or non-HN? Two of the HN traits involve sensitivity, and it's possible people perceived to have suffered pain (e.g., from a burn disfigurement) are seen as more sensitive (both emotionally and morally, as pain is often seen as ennobling people). The salience of their inferred suffering might lead to higher ratings on these items and thus relatively high HN scores. Ideally more attention might be paid post hoc to examining which HN and non-HN items showed differences between FD and other faces, as this might clarify the reasons for the surprising finding. In addition, it would have been desirable to have a longer and more desirable dehumanization measure and/or more than one measure.

**Response:** As advised by Reviewer 1, we conducted post-hoc to investigate the assumption that people with FD may be attributed more HN-traits because our HN-scale relies on two items measuring sensitivity. For that purpose, we conducted 3 repeated measures *t*-tests comparing attributions of each HN trait to targets with and without facial difference. We describe the results hereafter.

1. Attribution of comprehensiveness

Participants attributed more comprehensiveness to targets with FD than to targets without (*t*(96) = 4.53, *p* < .001, *d* = 0.46).

2. Attribution of emotional sensitivity

Participants attributed more emotional sensitivity to targets with FD than to targets without (*t*(96) = 3.03, *p* < .003, *d* = 0.31).

3. Attribution of moral sensitivity

Participants did not attribute more moral sensitivity to targets with FD than to targets without (*t*(96) = -0.92, *p* = .36, *d* = 0.09).

To conclude, these results confirm that people with FD are perceived as more sensitive but only in the emotional domain and not in the moral domain. Since these results also show that people with FD are perceived as more comprehensive, we cannot confirm that this scale was biased by an association between people with FD and sensitivity.

**Modifications**:

- Discussion: We draw the reader's attention to the need to move towards other, less desirable measures of dehumanization when it comes to measures of social perception. (p.10 & 11): “First, it is possible that the measures of dehumanization as a part of social perception cannot capture the dehumanization of people with FD, or at least the scale we have chosen.” And « More generally, measures to circumvent the social desirability bias would be advisable. »

3. Given that disgust was part of the rationale for expecting non-eye gaze might be associated with dehumanization, why was disgust not measured in the study?

**Response:** We agree with the reviewer that introducing disgust into this study would have had several benefits. First, it would have been an opportunity to replicate these results. Secondly, it would have allowed us to compare the effects on disgust with the effects on dehumanization. We could even have considered studying the mediating effect of disgust.

However, we did not introduce disgust for several reasons. Firstly, we would have had to considerably increase the number of participants, which was already substantial for an eye-tracking study. Secondly, in order not to present just one emotion (disgust), which could have influenced participants’ reactions, we would have had to add several emotions, thereby significantly increasing the procedure length.

Besides, studies are being currently underway to replicate these results on disgust. Finally, we had no precise hypothesis in terms of disgust mediation. Indeed, we first wanted to know whether there was a main effect of the presence of a facial difference on attributions of traits related to humanity.

4. It might also be worth noting on the Discussion that most work on trait attribution and dehumanization has focused on perceptions of groups rather than individuals, and when it has used individuals as targets it usually does not provide (humanizing) faces as stimuli. It is therefore not clear whether trait ratings of individual faces are an optimal way to assess dehumanization.

**Response:** We agree that our research, by providing participants with faces as stimuli, does not replicate much of the research conducted in the field of dehumanization, which has focused on intergroup relations. However, for example, Hugenberg and colleagues (2016, Study 3) did also use faces as stimuli.

**Modifications**:

- Discussion: We underline that faces as stimuli may have biased humanity attributions (p.11): “Second, we used faces displaying direct eye gaze, which may have biased humanity attributions. Faces are individuating stimuli [32]. Moreover, Khalid and colleagues (2016) [33] revealed that direct eye gaze signals an invitation to social interaction and thus promotes mind perception.”

# **Reviewer #2**

Thank you for inviting me to review this manuscript.
The paper describes an eye-tracking study investigating eye gaze towards the face of a person with a visible facial difference. The results replicated previous studies showing enhanced attention to the area of the visible difference but there was no relationship with measures of dehumanisation. The paper is written concisely and is mostly straightforward though the clarity could be improved in some places.

**Response:** For this new version of the document, we have completed the translation of the document with a new translation carried out by an external service in order to check the adequacy of the wording of the document with international standards. We hope this has improved the document's clarity.

NB: This initiative was also undertaken in response to the comments to authors 4 « Is the manuscript presented in an intelligible fashion and written in standard English? »

Major points

I’m not convinced that we should ever have expected that a person with a visible facial difference would be regarded as less human. Humanity is an attribution of the person, whereas the experiences of disgust mentioned in the introduction are a response to the visual appearance of the face, not the person. This distinction could be made more clearly.

The indirect findings referred to in the Introduction linking dehumanisation with visible facial difference did not amount to a compelling argument.

The Discussion seems determined to pursue the concept of dehumanisation in future studies but perhaps it would be more worthwhile to consider in what ways people with visible facial difference are regarded as different; dehumanisation may not capture the heart of how they are socially perceived.

**Response:** We appreciate the Reviewer's arguments but we still believe that the assumption of a dehumanization of visibly different people is grounded for several reasons.

First, we relied on research considering dehumanization in situations other than downplaying mind or characteristics (for a review, see Kteily & Landry, 2022). For instance, there is research showing that the disengagement in configural face processing is dehumanizing (Hugenberg et al., 2016). Thus, we hypothesized that people with facial difference may be dehumanized because there are evidences of a dehumanizing cognitive treatment of their face (see research cited in the section « dehumanization of people with facial difference » in our manuscript). Said differently, we hypothesized that people with facial difference would be dehumanized because they seemed to be treated differently than other human beings.

Second, the assumption that disgust is an emotional reaction to the face and not the person is not clearcut. Indeed, Shanmugarajah and colleagues (2012) revealed that people with facial difference elicit the different type of disgust, i.e., core disgust, contamination disgust and animal disgust. Moreover, recently, in a study that we thus did not include in our prior version of the manuscript, Landry and Schooler (2022) showed that the (intergroup) disgust felt toward people with facial difference predicted their dehumanization.

In any case, we agree with Reviewer 2 that dehumanization may not be the most appropriate concept to capture the public stigma of facial difference, and that our discussion should take this into account.

**Modifications**:

- Introduction: We added the findings of Landry and Schooler as a supplementary rational advocating for our hypothesis (p.4): “Since disgust is a precursor of dehumanization [19], including the dehumanization of people with facial differences [20], it is thus possible that this stigmatizing gaze could also be a dehumanizing gaze. »
- Discussion: We improved last paragraph (p.11): « Finally, it is possible that this stigmatizing gaze towards people with FD is not underpinned by dehumanizing attributions. Thus, other related – but independent – constructs, such as objectification [6], may better correspond to the lived experience of people with FD, which deserves future attention. Regardless, people with FD feel dehumanized (i.e., metadehumanization [35]), which may lead them to perceive themselves as diminished human beings (i.e., self-dehumanization [36]). Thus, the impact of this stigmatizing gaze on their self- and meta-dehumanization should also be investigated in future studies. »

Minor points
Page 5 – I could not follow the procedure without knowing how many faces, and how many versions of each face, were presented. The paper appears to have been written to a very tight word count, but even so, a minimal level of information should be included to assist the reader in understanding the research. I appreciate the information is in the supplementary materials, but it needs to be also (briefly) in the main paper.

**Response:** As Reviewer 2 pointed out, we wanted this document to be as concise as possible. We agree that we should have included more information about the stimuli.

**Modifications**:

- Method: More information was provided on the stimuli (p.6): “Eight faces were used as stimuli: four different faces presented either in their original condition or with an FD. Participants were randomly exposed to one of two sets of four faces. In each set, participants could see two faces with FD and two faces without FD; each time, one face belonged to a woman, and the other to a man. For the two faces presenting an FD, one of them had an FD pattern on the left cheek, while the other had an FD on the right cheek. Each face was presented either in its original or in its “disfigured” condition depending on the set of faces.”

Page 6 – similarly, UH traits and HN traits should be defined, albeit briefly, in the main paper.

**Response:** In line with Reviewer 2’s recommendation, we provide more information about UH and HN characteristics.

**Modifications**:

- Introduction: We provide a short definition for UH and HN traits (p.3): “More precisely, people can be dehumanized when they are denied uniquely human (UH) characteristics (e.g., refinement; i.e., characteristics that distinguish human beings from nonhuman animals) and/or characteristics that are at the essence of human nature (HN; e.g., moral sensitivity; i.e., characteristics that distinguish human beings from inert entities) [8,9].”

I didn’t understand this point, so please rephrase “Two overarching manifestations of dehumanization have been described: dehumanization as a content of social perception (e.g., attribution of nonspecific human characteristics), and dehumanization as a form of perception (7).”

**Response:** We apologize for the lack of clarity. We did our best to improve this sentence.

**Modifications**:

- Introduction: We rephrased the sentence (p.3): “Two broad types of dehumanization manifestations have been described: dehumanization as the content of social perception (e.g., the attribution of nonspecific human characteristics) and dehumanization as a form of perception (i.e., the reduction of people to their constituent parts) [6].”
