# Supplementary material for: Only human after all? a pre-registered study on gaze behavior and humanity attributions to people with facial difference
Source: PLoS One. 2023 Dec 12;18(12):e0295617. doi: 10.1371/journal.pone.0295617 (PMC10715648; doi:10.1371/journal.pone.0295617)
Supplement: Attachment — Submitted filename: Response to Reviewers.docx [file pone.0295617.s003.docx]

Submission of the revised version of a paper in “PLOS ONE”

Responses to Reviewers

PLOS ONE
Ref: PONE-D-23-17359

November, 14th, 2023

# **Editor**

***The manuscript is very close to be ready for acceptance. However, I think the manuscript would further benefit from including some of the discussion and response you have given to reviewer #1's comment about the lack of inclusion of a disgust measure in the study. I feel this is a valid question and one that other readers are likely to ask, so including your response to the reviewer's comment somewhere in the manuscript (perhaps in the Discussion?) would be useful. I hope this is not too onerous, and I think this addition would ensure the manuscript can be accepted for publication.***

**Response:** In line with your recommendation, we discuss the lack of inclusion of a disgust measure.

**Modifications**:

- Discussion: We discussed the rationale for not including a disgust measure and its implications (p.10-11): « In this research, we did not include a measure of disgust for theoretical (e.g., we were concerned that priming our participants with disgust might bias their visual attention) and technical reasons (e.g., a longer procedure might have exhausted participants – this would have been particularly true in the case of including multiple affects). However, given that previous research on disgust [20] led us assume a dehumanization of people with FD that was not supported by our data, the impact of disgust on social perceptions of people with FD would merit future attention. »
